# Phenotypic Plasticity, Epigenetic or Genetic Modifications in Relation to the Duration of Cd-Exposure within a Microevolution Time Range in the Beet Armyworm

**DOI:** 10.1371/journal.pone.0167371

**Published:** 2016-12-01

**Authors:** Maria Augustyniak, Anna Płachetka-Bożek, Alina Kafel, Agnieszka Babczyńska, Monika Tarnawska, Agnieszka Janiak, Anna Loba, Marta Dziewięcka, Julia Karpeta-Kaczmarek, Agnieszka Zawisza-Raszka

**Affiliations:** 1 Department of Animal Physiology and Ecotoxicology, University of Silesia, Katowice, Poland; 2 Department of Genetics, University of Silesia, Katowice, Poland; Chinese Academy of Agricultural Sciences Institute of Plant Protection, CHINA

## Abstract

In the case of the pests inhabiting metal polluted or fields where the use of pesticides is common, a natural selection of resistant individuals can occur. This may pose serious problems for humans, agriculture, as well as the economies of many countries. In this study, the hypothesis that multigenerational (120 generations) exposure to cadmium of a beet armyworm population could be a selecting factor toward a more efficient DNA protection was verified. The hemocytes of individuals from two culture strains (control and Cd-exposed) were treated with H_2_O_2_ (a DNA-damaging agent) or PBS (reference). The level of DNA damage was assessed using the Comet assay immediately and 5, 15 and 30 min. after the treatment. The immediate result of the contact with H_2_O_2_ was that the level of DNA damage in the hemocytes of the insects from both strains increased significantly. However, in the cells of the Cd-exposed individuals, the level of DNA damage decreased over time, while in the cells from the control insects it remained at the same level with no evidence of repair. These results suggest that efficient defense mechanisms may exist in the cells of insects that have prolonged contact with cadmium. Some evolutionary and trade-off aspects of the phenomenon are discussed. In a wider context, comparing the results obtained in the laboratory with field studies may be beneficial for understanding basic mechanisms of the resistance of an organism. To summarize, the high potential for the repair of DNA damage that was observed in the insects from the cadmium strain may confirm the hypothesis that multigenerational exposure to that metal may possibly contribute to the selection of insects that have a wider tolerance to oxidative stress. However, our investigations of polymorphism using AFLP did not reveal differences between the two main insect strains.

## Introduction

The connections between laboratory investigations and field research on pest insects create serious problems for science today. The defense mechanisms that are investigated in laboratory conditions usually do not fully explain the differences between natural populations of pests that have been exposed to various pollutants for many years. On the other hand, results derived from field populations are very difficult to interpret because of the number and complexity of processes, factors and their interactions the populations are exposed to. It is hard, or even impossible, to evaluate and assess their impact on the functions of an organism. Investigation of the defense mechanisms in laboratory-bred organisms selected for a sufficient time toward tolerance to stressors could be a good solution. Such a procedure can serve as a good, simplified model, which can simulate the processes that exist in natural conditions. It is important, however, to stress that long-term exposure of animals to a particular stressor might lead to the selection of individuals that cope better with this stressor in the same manner as in their natural environment. This phenomenon is of particular (and also practical) importance in the case of the insect species that are usually recognized as a dangerous, highly expansive crop pests, e.g. *Spodoptera exigua* [1–3].

Beet armyworm, *Spodoptera exigua* Hübner (Lepidoptera: Noctuidae), which originates from Southern Asia, is one of the best-known agricultural pests. It is known to feed on over 50 plant species. In the 19^th^ century, it was introduced to the United States. Today, *S*. *exigua* occurs worldwide and can be found anywhere that its host crops are grown [3,4]. Irrespective of the development of new methods to control these pests such as sex pheromones or insect-resistant transgenic crops, farmers still have to use pesticides. Additionally, in various parts of the world, these insects have lived under long-term pollution pressure resulting from industrialization and the development of human civilization. This may lead to the development of resistance (or so-called cross-resistance) to various xenobiotics. This is why scientists around the world are extremely interested in studying *S*. *exigua* [1–3,5,6]. A unique strain of *S*. *exigua* has been bred in our laboratory. In 2001, we selected a cadmium strain from among a maternal culture of the insects. The insects of each generation within this strain have been fed food contaminated with cadmium (44 μg·g^-1^ dry weight of food). To date, we have obtained over 120 generations of cadmium-exposed beet armyworms. There are serious reasons to believe that the insects from this strain can react to additional stressing factors differently [7,8] compared to the original, Cd-unexposed strain.

The existence of an animal under permanent, mild stress (e.g. caused by contact with a sublethal dose of metal) requires a trade-off. An increase in the protective and/or repair protein synthesis, which allows the negative effects of harmful substances to be minimized and the lifespan to be prolonged, is beneficial for an individual. Natural selection can occur in the case of pests inhabiting areas polluted with metals or fields where the use of pesticides is common. This is of key importance for agriculture and the economies of many countries. The time that is required for a change in gene frequency or for a genetic adaptation (in the strict sense) to become effective depends on the duration of generation time and phenotypic plasticity to survive the change. Taxa that have a rapid succession of generations are less dependent on physiological flexibility and, at the same time, are more susceptible to changes in gene frequency in populations. In the case of insects, such modifications of a genome can last for years [9]. However, long-term contact with a xenobiotic does not have to lead to changes that become genetically preserved. Moreover, mechanisms such as individual phenotypic plasticity, epigenetic modifications, maternal effects and other forms of nongenetic inheritance can underlay the adaptive responses of organisms to changing environmental conditions and can be very important [10–12].

In this study, we attempted to answer the question: does long-term (for 120 generations) contact of *S*. *exigua* individuals with cadmium contribute to the selection of insects that have better defensive strategies compared to unexposed insects? The questions of DNA stability and susceptibility to DNA damage of insects from such a unique, preselected strain is especially important in order to understand how the mechanisms of resistance evolve in insects. The experiments that were performed during this study enabled us to test the following, alternative hypotheses:

1. The amount of DNA damage (DNA strand breaks) in the hemocytes of insects from the cadmium strain will not significantly differ compared to those from the reference group. The basal level of damage in both strains is typical for the species because the maintenance of genome stability is a priority for species survival.
2. The amount of DNA damage in hemocytes of insects from the cadmium strain will be lower than in the reference insects. This phenomenon may be associated with some form of hormesis or contact with cadmium that lasts for 120 generations, which can cause the selection of individuals that can effectively protect genetic material and/or effectively repair the damage.
3. The level of DNA damage in the hemocytes of the beet armyworms from the cadmium strain will be higher than in the insects from the reference strain. This may indicate a genotoxic action of cadmium and/or a dysfunction in the repair mechanisms, which could result in a lack of tolerance symptoms due to the prolonged contact of individuals with cadmium.

To test the hypothesis, we chose the Comet assay (SCGE—Single Cell Gel Electrophoresis), a good, sensitive and relatively fast marker of exposure to various xenobiotics. It allows both single- and double strand breaks to be detected and the repair potential to be assessed [13–18]. Moreover, this technique has previously been applied by other investigators to determine whether metals such as Cu, Pb, Ni, Cd, Co, Zn can generate breaks of DNA strands [14,19–21]. However, studies on DNA stability in insects selected toward a resistance to metals in laboratory conditions (i.e. cadmium) for over a dozen years have not been conducted so far. The study is novel in this approach and provides the opportunity to see the problem of resistance development in crop pests in a new light.

DNA fingerprinting with molecular markers, such as for example Amplified Fragment Length Polymorphism (AFLP), allows to asses genetic differentiation between individuals and to describe them in terms of multilocus genotypes (MLG)–the composition of alleles derived from observed AFLP band pattern. Once sufficient genetic diversity is detected, it is possible to search for associations between MLG and phenotypic observations. This in turn may allow to distinguish between genetic and nongenetic nature of adaptive responses and to point out to the possible importance of specific MLGs in the emergence of particular phenotype (genetic inheritance) or to the phenotypic plasticity underlying the diversity of the traits under study [22,23]. AFLPs may also be used in the study of microevolution processes by the analysis of gene frequencies, occurrence of mutations, detection of bottleneck effect, gene flow or inbreeding [24]. It may also serve to detect the proportion of genome that is the subject of divergent selection and to dissect the patterns of neutral and adaptive divergence [25,26].

## Materials and Methods

### Animal breeding

The individuals used in the experiment originated from laboratory breeding in which beet armyworms (*Spodoptera exigua*) have been reared since 1999. The cadmium strain was established more than ten years ago. Currently, animals that have been treated with cadmium (44 μg Cd per g of dry mass of food) for 120 generations are available.

Larvae were fed with an artificial diet that contained a mixture of wheat germ, yeast powder, casein, sucrose, Wesson salt mixture, Vanderzant vitamin mixture, streptomycin sulfate, formaldehyde, methyl p-hydroxybenzoate, agar and water. The food was given *ad libitum*. The temperature of the breeding laboratory (25 ± 1°C) and a light:dark cycle of 16:8 hours were maintained. Larvae and pupae were kept in Petri dishes. Moths were kept in plastic cages (volume 2.5 L) with access to a water solution of honey (10% v/v) as food.

The experiments were conducted using the insects from the original control (C) and cadmium (Cd) strains. In the original strains, cadmium was added to the rearing medium for 120 generations, while the control group was reared on a medium without cadmium (variant 0). In variant 1, the control insects were transferred to the cadmium-containing food (C→Cd) and the insects from the cadmium strain were transferred to cadmium-free food (Cd→C) for a short-term exposure (one week). In variant 2, the insects were fed as in variant 1, but the exposure lasted for one generation.

An apoptosis profile in hemolymph samples and a comet assay were performed in all three variants. The concentration of hydrogen peroxide in the hemolymph samples was measured in the insect hemolymph in the case of variants 0 and 2 while AFLP polymorphism was determined for the insects in the case of variant 0.

### Apoptosis profile of hemocytes

Insects of the 5^th^ instar were randomly selected in all three variants (12 individuals in variant 0, and 6 individuals in variants 1 or 2) from each strain. The insects were anesthetized on ice and samples of the hemolymph (40 μL) were collected (see the description below) and mixed with an anticoagulant buffer at a 1:1 ratio.

All of the measurements were performed using a Muse^®^ Cell Analyzer (Muse^™^ Cell Analyzer; Millipore, Billerica, MA, USA) flow cytometer according to the manufacturer’s protocols after standardization for the *S*. *exigua* hemolymph. The total number of cells was measured using the Muse^™^ Count & Viability test. The average number of cells in the suspension was set in a range 10^5^ to 10^6^ cells/mL. The apoptosis profile was examined using a Muse^™^ Annexin V and Dead Cell assay, which is based on the detection of phosphatidylserine (PS) on the surface of apoptotic cells.

### H_2_O_2_ assay

The H_2_O_2_ in the hemolymph samples as well as in the reference samples (PBS only with H_2_O_2_, which was added as described below) was detected using an Ampliflu^™^ Red reagent (Sigma, 90101) according to [27]. The H_2_O_2_ concentration was measured in the samples collected from the insects in variants 0 and 2.

The amount of total hemolymph collected from the 5^th^ midstage larvae (40 μL) was dissolved in a PBS buffer pH 7.4 (460 μL). Then, each hemolymph sample was measured after mixing equal volumes H_2_O_2_ (to obtain a final concentration equal to 50 μM) or with PBS. One hundred μL of the reaction mixture consisted of 50 μL of the hemolymph samples and 50 μL of the compound of Ampiflu red reagent (50 μM) with horseradish peroxidase (0.1 U/mL) in a 0.05 M phosphate buffer pH 7.4. The mixture was incubated at room temperature in darkness for 30 minutes. After incubation, the absorbance was measured at 576 nm using a microplate reader (Infinite M200, Tecan). The μmol amounts of H_2_O_2_ were calculated according to a standard curve obtained by the incubation of the H_2_O_2_ solutions in the range of 0.05 to 5 μmol. Measurements were repeated at different time intervals: immediately after hemolymph collection (time 0) and 20 or 30 minutes after hemolymph incubation with H_2_O_2_.

### The Comet assay

#### Cell collection and measurement set-up

The level of DNA damage was measured in hemolymph cells that had been collected from the 5^th^ instar of *S*. *exigua*. Individuals from both strains were first slightly anesthetized on ice and then one proleg of the caterpillar was cut off. A drop of the hemolymph leaking from the incision (40 μL) was collected into Eppendorf tubes that had previously been filled with 460 μL of a phosphate buffer (pH 7.4). A hemolymph suspension prepared this way was then divided into two equal parts, 250 μL each. In the first part, 12.5 μL of the suspension was substituted with a H_2_O_2_ solution (50 μM final concentration) to induce DNA damage. In the reference group (second part of cell suspension), 12.5 μl of the suspension was substituted with a phosphate buffer. All of the samples were incubated for 1 min to induce DNA damage [28]. Then, 50 μL of the cell suspensions from treated and reference samples were transferred onto prepared slides immediately (0 min) to assess the DNA damage, and 5, 15 and 30 min after the incubation period to assess the DNA repair. The assessments were carried out using the comet assay (see below). The procedure was repeated independently five times for insects from the control and cadmium strains (40 slides per each strain) in all three variants of the experiment. A total of 240 slides was prepared and analyzed.

#### Slide preparation and analysis

The comet assay was performed under alkaline conditions according to the procedure described previously [29]. Briefly, the cell suspension (50 μL), which had been kept on ice and in darkness, was mixed with 50 μL of 1.5% low melting point agarose (LMPA; 1:1, v/v), spread onto slides covered with 1% normal melting point agarose (NMPA) and again covered with 1% LMPA agarose as the top layer. Next, the slides were immersed in a lysis buffer (2.5 M NaCl, 100 mM EDTA, 10 mM Tris, 0.25 M NaOH, 1% TritonX-100 and 10% dimethyl sulfoxide (DMSO), pH 10.0) for 2 h at 4°C. Pre-electrophoresis and electrophoresis (0.3 A) were performed in a horizontal gel electrophoresis unit (Comet DNA set 2x10 with thermal stabilizer, Kucharczyk, Poland) in a buffer consisting of 300 mM NaOH and 0.9 mM Na_2_EDTA, pH>13 for 20 min each. After neutralization (0.4 M Tris-HCl; pH 7.4), the slides were fixed with methanol and dried overnight at room temperature. Before the analysis, slides were moistened with _d_H_2_O, dyed with 0.1% DAPI and visualized under a fluorescence microscope (Nikon Eclipse 80i with B&W Video Camera SONY) with a Komet 5.5 image analysis system (Kinetic Imaging, Liverpool, UK). Tail DNA (TDNA), tail length (TL) and olive tail moment (OTM) were measured for 50 randomly selected nuclei on each slide.

### AFLP polymorphism

#### DNA isolation

Genomic DNA was extracted from the head and thorax of individual larvae from both breeding strains (Variant 0). A GeneMATRIX Tissue DNA Purification Kit was used for the isolation and purification of genomic DNA. DNA samples were subjected to electrophoresis to confirm their quality and quantity.

#### AFLP analysis

Five individuals of *S*. *exigua* from the control and cadmium breeding strains were used to test their polymorphism at the DNA level by the AFLP markers. Restrictions were processed in 10 μL of a restriction solution containing 250 ng of genomic DNA, 1.25 units of each: *MseI* and *EcoRI* enzymes (New England BioLabs) for three hours at 37°C followed by enzyme inactivation at 70°C for 10 minutes. The adaptor-ligation solution contained 0.6 units of T4 DNA ligase (MBI Fermentas), 3 pmol of EcoRI adaptor and 30 pmol of MseI adaptor. The ligation reaction lasted for 16 hours at 37°C. Preselective amplification was performed in 9.2 μL of a reaction mixture containing 0.5 μL of the restriction and ligation products, 7.5 ng each of Eco+A and Mse+C primers, 200 μM of dNTPs (Amersham Pharmacia), 0.1 U of Taq polymerase (DyNAzyme, Finnzymes) and a 1 x PCR buffer (10 mM Tris-HCl (pH 8.8 at 25°C), 1.5 mM MgCl_2_, 50 mM KCl and 0.1% Triton X-100). The PCR conditions were 20 cycles of 30 s at 94°C, 40 s at 56°C and 50 s at 72°C. Next, the samples were dissolved ten times before selective amplification, which was carried out in an 8 μL volume with 2.5 μL of the preselective amplification product and 5.5 μL of the selective amplification solution (0.6 pmol of the EcoRI primer labeled with fluorescent dye IRD 800, 15 ng of MseI primer, 250 μM of dNTPs (Amersham Pharmacia), 0.25 U of Taq polymerase (DyNAzyme, Finnzymes), 2.1 mM of MgCl_2_ and a 1 x PCR buffer (10 mM Tris-HCl (pH 8.8 in 25°C), 1.5 mM MgCl_2_, 50 mM KCl and 0.1% Triton X-100). The touchdown PCR protocol for the selective amplification consisted of two phases—phase 1 included an initial step of 94°C for 30 s, followed by 12 cycles of denaturation at 94°C for 30 s, annealing at 68°C (decreasing by 0.7°C in each cycle, up to 55.6°C) for 30 s and extension at 72°C for 80 s. Phase 2 consisted of 23 cycles of 94°C for 30 s, 59°C for 30 s and 72°C for 1 min. The following primer pairs were used in the selective amplification step: E-AAG/M-CAA, E-AG/M-CAA, E-AA/M-CAA, E-AT/M-CAA, E-ACC/M-CAA, E-ACC/CAG, E-ACC/M-CTA, E-ACC/M-CAT, E-ACC/M-CAC, E-AC/M-CAC, E-AC/M-CAG, E-AC/M-CTA. After the PCR reaction, 4 μL of a formamide gel loading buffer was added to the samples. The visualization of the products was performed in denaturing polyacrylamide gels (6% acrylamide/bis-acrylamide 19:1 solution (Sigma), 7 M urea (Amersham Pharmacia), 1x TBE) using a Li-Cor 4300 automated sequencer. The following conditions were used for the electrophoresis: 1300 V, 30 mA, 30 W. Medium speed laser scanning was used. The size of the amplified fragments that had fluorescent signals was determined using the 50–350 bp IRDye800 Concentrated Sizing Standard (Li-Cor).

#### AFLP data analysis

The level of AFLP polymorphism was defined in two ways—as the percentage of the variable loci over the total number of loci and as a pairwise comparison of the percentage of polymorphic bands between each pair of the individuals studied. The range of pairwise polymorphism was given and the mean pairwise polymorphism was calculated as: P = Σ (Aij/Bij × 100)/N; where: A—the number of polymorphic loci for a pair of samples *i* and *j*, B—the total number of loci for a pair of samples analyzed and N—the number of pairs of samples analyzed.

### Statistical procedures

The profile of apoptotic changes was analyzed for 12 samples in the case of variant 0 and for six samples in the case of variants 1 and 2. The concentration of hydrogen peroxide in the hemolymph was measured in six replicates. The means and standard deviations were calculated in each group. The significance of differences was determined using Tukey test (p < 0.05).

For the comet assay, five slides were analyzed in each experimental group. The means and medians were calculated for all three parameters for each slide. These data were then subjected to statistical analysis in order to find any differences between the experimental groups. The normality of distribution and homogeneity of variance were checked using commonly accessible tests (the Kolmogorov-Smirnov and Shapiro-Wilk tests, as well as the Levene test). The median values that were determined for each slide were used in further analyses. The Tukey test (p < 0.05) was used for the insects from each strain separately to check the differences in the damage level at consecutive time points. A generalized linear model was used to assess the main effects (GLM, p < 0.05; only for variant 0). Statistical analysis was performed using the STATISTICA 10.0 software package (StatSoft, Inc. (2010).

## Results

### Cellular apoptosis of hemocytes

**Fig 1:**
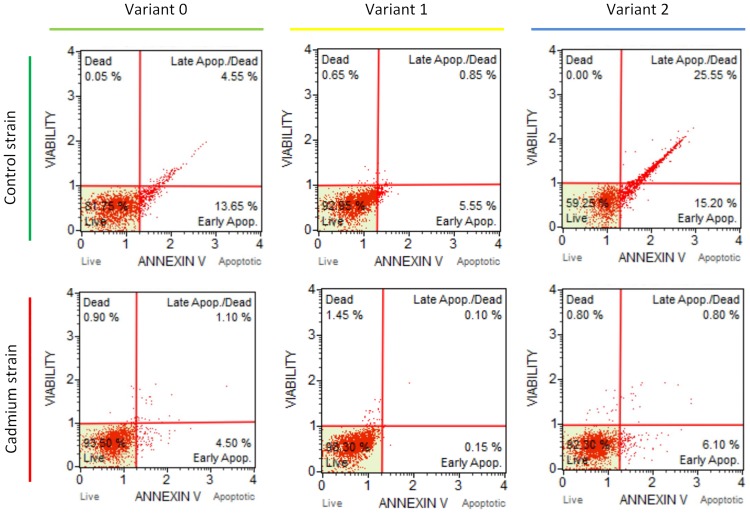
Apoptosis profile in exemplary hemolymph samples of the 5^th^ instar of *Spodoptera exiqua* from the control and cadmium strains as measured by the Muse^™^ Annexin V and Dead Cell assay. Abbreviations: Variant 0 –individuals from the control and cadmium-treated strains through 120 generations; Variant 1 –individuals from the control and cadmium strains that were fed the exchanged diet (control to cadmium or cadmium to control) for one week. Variant 2 –individuals from the control and cadmium strains that were fed the exchanged diet for one generation. The lower left (LL) quadrant represents normal healthy cells; the lower right (LR) and upper right (UR) quadrants represent early and late apoptotic cells, respectively; the upper left (UL) quadrant represents necrotic cells.

**Fig 2:**
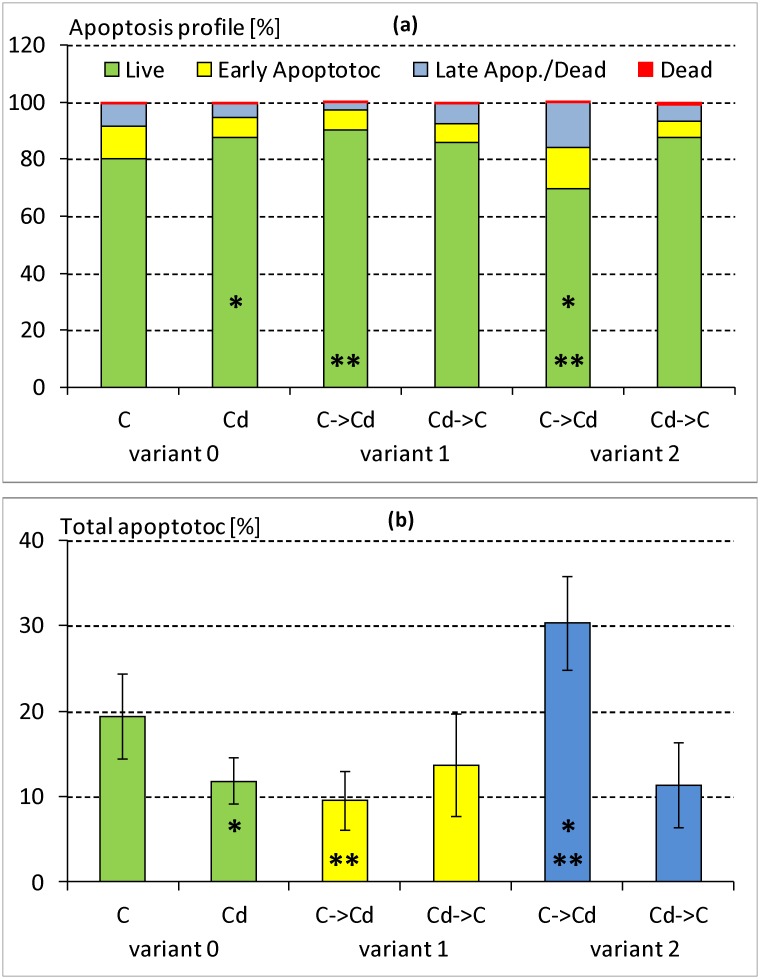
Apoptosis profile (a)–%; mean of 12 or 6 measurements in variant 0 or variants 1 and 2, respectively and total apoptotic cells (b)–mean ± SD in the hemolymph samples of the 5^th^ instar of *Spodoptera exiqua* from the control and cadmium strains. Abbreviations: Variant 0 –individuals from the control (C) and cadmium-treated (Cd) strains through 120 generations; Variant 1 –individuals from the control and cadmium strains that were fed the exchanged diet (control to cadmium (C->Cd) or cadmium to control (Cd->C)) for one week. Variant 2 –individuals from the control and cadmium strains that were fed the exchanged diet for one generation. Stars denote statistically significant differences between the C->Cd group in variant 2 and the C->Cd group in variant 1 or the Cd group in variant 0 (ANOVA, Tukey test, p < 0.05).

The profile of the apoptosis was measured in the hemolymph of the 5^th^ instar larvae in all three variants of both strains (Figs 1 and 2). In the variant 0 a slightly higher percentage of living cells in the insects from the Cd strain compared to the control strain insects was found. This difference, however, was not statistically significant. Moreover, no statistically significant differences between the strains were found within variants 1 and 2. The exposure of control insects to cadmium-containing diet for one generation appeared to cause a tendency to decrease the percentage of living cells in the hemolymph compared to the remaining insect groups that had been exposed to cadmium (Cd-exposed for one week and Cd-exposed for 120 generations). The number of total apoptotic cells in this case was high and reached 30% (Fig 2b). This value was significantly higher compared to the one found in the insects from the control strain, which had been transferred to the Cd-containing food for one week (variant 1) or compared to the insects that had been exposed to multigenerational Cd exposure (variant 0).

### Concentration of H_2_O_2_ in the hemolymph samples

**Fig 3:**
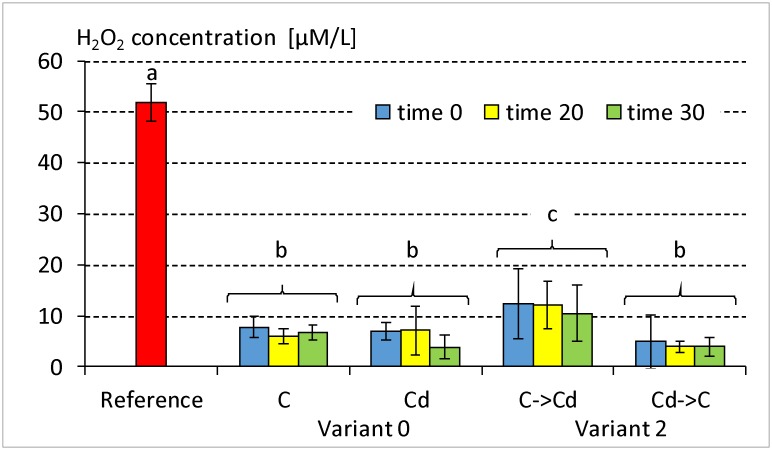
Mean ± SD concentration of hydrogen peroxide in the hemolymph samples of the 5^th^ instar of *Spodoptera exiqua* from the control and cadmium strains. Abbreviations: Reference—concentration of H_2_O_2_ in the samples without hemolymph, Variant 0 –individuals from the control (C) and cadmium-treated (Cd) strains through 120 generations; Variant 2 –individuals from the control and cadmium strains that were fed the exchanged diet for one generation. The same letters indicate homogenous strain-groups (ANOVA, Tukey test, p < 0.05).

The actual content of H_2_O_2_ in the reference samples and in the samples that had been incubated with H_2_O_2_ in variants 0 and 2 were determined. The content of H_2_O_2_ in the reference sample, which had been prepared in the same way as the experimental samples but without hemolymph, was equal to 51.8 μM/L and was significantly higher than in the case of hemolymph containing samples (Fig 3). This value was almost identical as the final concentration that was expected to be obtained after mixing the hemolymph samples with the H_2_O_2_ solution to induce DNA damage.

Only the mean values of the H_2_O_2_ concentrations in the samples of the hemolymph of the insects that had been transferred to the Cd-containing diet for one generation (variant 2) were significantly higher than in the remaining experimental groups. This result corresponded to the profile of apoptosis (Figs 1 and 2). No significant time-dependent differences were found in the H_2_O_2_ concentration.

### The results of the comet assay

#### Variant 0 –original strains

**Fig 4:**
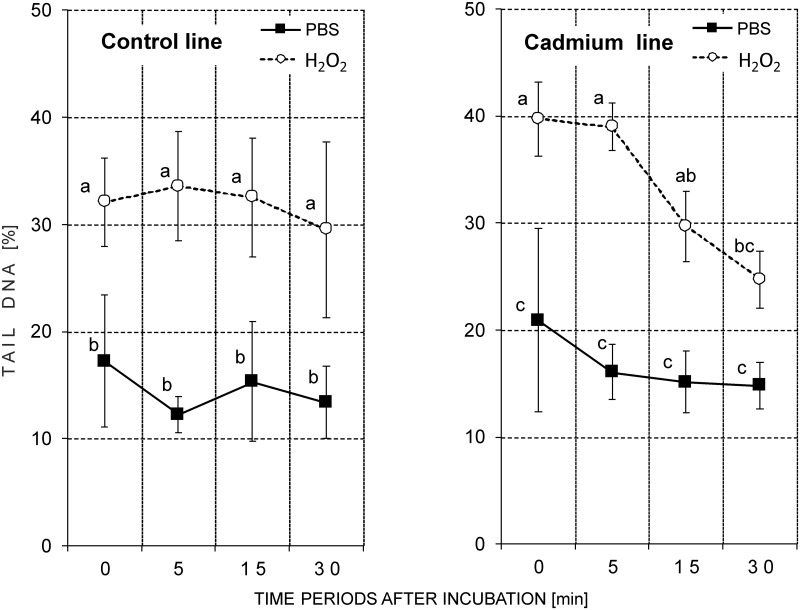
Tail DNA (%; mean ± SD) in the nuclei of the hemocytes of the 5^th^ instar of *S*. *exigua* from the control and cadmium strains (Variant 0). After isolation, the cells were suspended in PBS and mixed with a H_2_O_2_ solution (treated groups; final concentration 50 μM) or with PBS (reference groups) and incubated for 1 min. Abbreviations: ○ or ■ –mean of the medians of fifty nuclei that were measured on each slide; 0, 5, 15 or 30 min—time period after the end of the incubation; the same letters indicate homogenous groups within a strain (ANOVA, Tukey test, p < 0.05).

**Fig 5:**
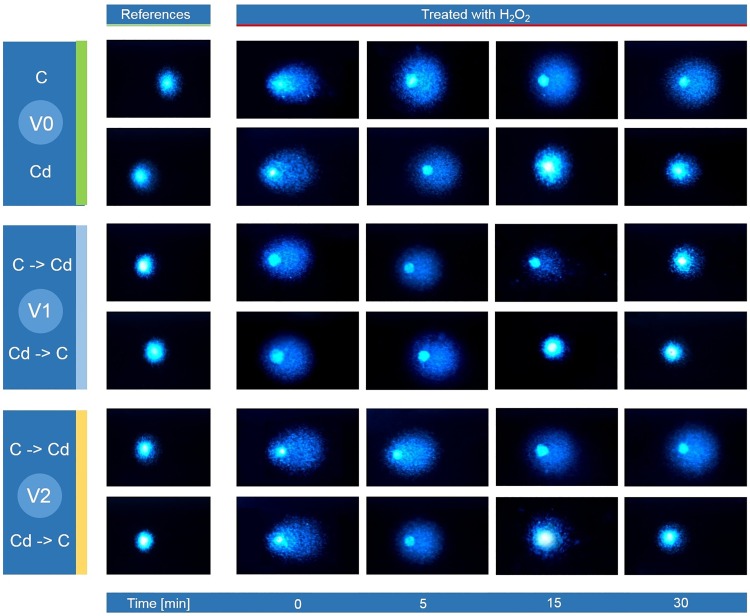
DNA comet images in the hemocytes of the 5^th^ instar of *S*. *exigua* from the control and cadmium strains. After isolation the cells were suspended in PBS and mixed with a H_2_O_2_ solution (treated groups; final concentration 50 μM) or with PBS (reference groups) and incubated for 1 min. Abbreviations: Variant 0 –individuals from the control and cadmium-treated strains through 120 generations; Variant 1 –individuals from the control and cadmium strains that were fed the exchanged diet (C->Cd: control to cadmium or Cd->C: cadmium to control) for one week. Variant 2 –individuals from the control and cadmium strains that were fed the exchanged diet for one generation; Time—time period (0, 5, 15 or 30 min) after the end of the incubation with H_2_O_2_ or PBS.

In all of the analyzed slides, the nuclei with varying DNA damage levels were found in the cells from both the control and H_2_O_2_-treated groups. The highest level of damage was found in cells immediately after exposure to H_2_O_2_. The amount of DNA damage decreased at the subsequent time points, but the values of parameters studied were different in the control and cadmium strains (Figs 4 and 5; Table 1; S1 Fig).

**Table 1:** DNA damage (mean ± SD) in the nuclei of the hemocytes of the 5^th^ instar of *S*. *exigua* from the control and cadmium strains (Variant 0). After isolation the cells were suspended in PBS and mixed with a H_2_O_2_ solution (treated groups; final concentration 50 μM) or with PBS (reference groups) and incubated for 1 min.

Parameter	TDNA [%]					TL (μm)					OTM (arbitrary units)				
	Control strain			Cadmium strain		Control strain			Cadmium strain		Control strain			Cadmium strain	
Time interval	M	SD		M	SD	M	SD		M	SD	M	SD		M	SD
*PBS (reference groups)*															
0 min	17.26	6.15		20.92	8.58	7.46	2.01		6.82	2.96	1.66	0.77		1.71	1.09
5 min	12.29	1.70		16.08	2.59	4.43	1.06		6.15	1.86	1.02	0.22		1.35	0.23
15 min	15.34	5.59		15.14	2.87	6.12	2.83		5.02	1.45	1.29	0.70		1.11	0.36
30 min	13.38	3.38		14.82	2.17	4.69	2.83		6.37	0.79	1.00	0.30		1.33	0.34
*H*_*2*_*O*_*2*_ *(treated groups)*															
0 min	32.11	4.11	*	39.71	3.44	14.80	2.86		14.59	1.56	3.65	0.60	*	4.54	0.44
5 min	33.58	5.11	*	38.95	2.21	12.56	2.50		12.91	1.24	3.86	0.65		4.16	0.35
15 min	32.53	5.55		29.69	3.27	13.26	2.79	*	10.24	1.43	3.79	0.85	*	3.17	0.43
30 min	29.52	8.19	*	24.70	2.65	11.92	3.10	*	9.51	1.04	3.32	0.94	*	2.69	0.28

Abbreviations: TDNA—Tail DNA; TL—Tail Length, OTM—Olive Tail Moment; M—mean, SD—standard deviation, *–indicates significant differences between the control and cadmium lines for specific time period (0, 5, 15 or 30 min) at the end of the incubation with H_2_O_2_ (ANOVA, Tukey test, p < 0.05; n = 5 slides with 50 nuclei each in each group).

Among the groups of cells that had been incubated in PBS, the basic DNA damage level was slightly higher in the cadmium strain compared to the control strain (Table 1). TDNA in the control strain was between 12.3%-17.3%, and it was between 14.8%-20.9% in the cadmium strain (Fig 4; groups treated with PBS). The value of the tail length (TL) in the reference groups was low and was similar in both strains. TL was between 4.4 μm-7.5 μm (S1 Fig; groups treated with PBS). A statistical test did not show any significant differences in the basic DNA damage levels between the control and cadmium strains (Table 1; Fig 4 –reference groups; S1 Fig).

Immediately after H_2_O_2_ treatment, the amount of DNA in the comet tail increased noticeably in both strains compared to the reference groups. The TDNA value (39.7%) was significantly higher in the hemocytes that had been isolated from the cadmium-strain individuals comparing to the insects in the control strain (32.11%). Tail length also increased after H_2_O_2_ treatment and reached values near 15 μm at the first time point (Table 1).

All three analyzed parameters remained at the same level in the hemocytes of the individuals in the control strain for the entire duration of the experiment, that is for 30 minutes after the end of cell incubation with H_2_O_2_ (Fig 4; S1 Fig). This means that a significant difference between the reference and treated groups was maintained until the end of the experiment in the control strain (Fig 4; S1 Fig).

Unlike in the control strain, a decrease in the level of DNA damage occurred over time in the cadmium strain (Fig 4 –right side of figure; S1 Fig). Fifteen minutes after H_2_O_2_ treatment, a significant decrease of TL and OTM was observed compared to the group that was measured at the first time point. For the cadmium strain, no significant differences between the samples of H_2_O_2_- and PBS-incubated cells were found 30 minutes at the end of the incubation (Fig 4; S1 Fig).

Main effect analysis (GLM, p < 0.05) indicated a significant influence of the “treatment” and “time” variables on all three of the DNA damage parameters analyzed. Although the “strain” variable had a significant influence on TDNA and TL, it did not change the OTM values noticeably (Table 2).

**Table 2:** The analysis of the main effects (General Linear Models—GLM; p<0.05) for the three parameters of the comets (TDNA, TL, OTM) that were obtained from the hemocytes of the 5^th^ instar of *S*. *exigua* from the control- and cadmium-treated strains.

Source of variation	*d*.*f*.	TDNA		TL		OTM	
		*F*	*p*	*F*	*p*	*F*	*p*
Strain (S)	1	12.97	0.0003	7.90	0.0050	0.99	0.3205
Treatment (Tr)	1	1216.04	<0.0001	1682.12	<0.0001	1539.25	<0.0001
Time (T)	3	36.54	<0.0001	57.22	<0.0001	33.700	<0.0001
(S) x (Tr)	1	0.76	0.3831	29.16	<0.0001	1.53	0.2154
(S) x (T)	3	16.12	<0.0001	15.44	<0.0001	11.57	<0.0001
(Tr) x (T)	3	15.17	<0.0001	12.31	<0.0001	11.70	<0.0001
(S) x (Tr) x (T)	3	5.40	0.0010	8.39	<0.0001	10.08	<0.0001

Abbreviations: Categorical factors: strain—S (insects treated with cadmium versus untreated control insects); treatment—Tr (cell suspensions treated with H_2_O_2_, or with H_2_O); time—T (comet analysis performed 0, 5, 15 and 30 minutes after H_2_O_2_ exposure).

The joint effect of the “strain” and “treatment” variables had an insignificant influence on the levels of DNA damage in the hemocytes of the larvae from both strains. TL was the only one that was significantly affected. Analysis of the joint effect of all three categorical variables (“strain”, “treatment” and “time”) indicated the influence of these variables on all three parameters (Table 2).

#### Variant 1 –insects on the exchanged diet for one week

**Fig 6:**
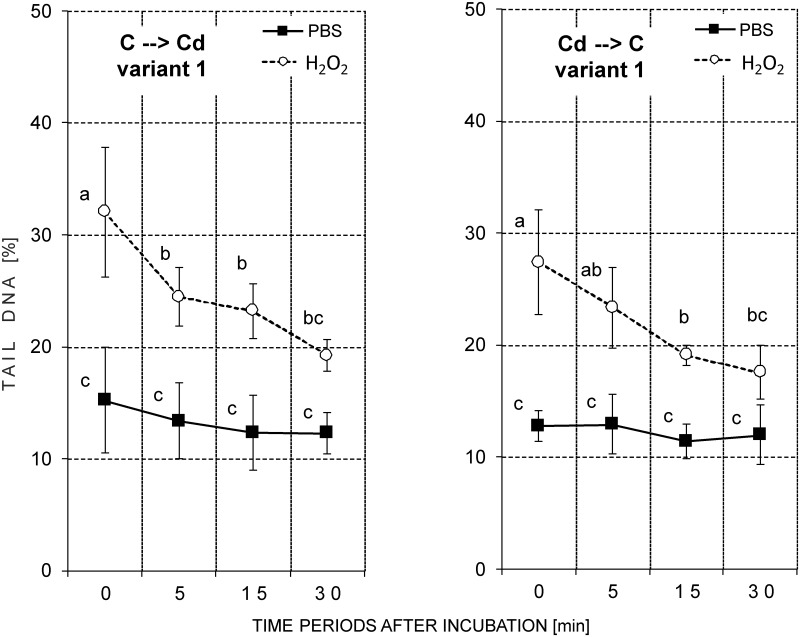
Tail DNA (%; mean ± SD) in the nuclei of the hemocytes of the 5^th^ instar of *S*. *exigua* from the control and cadmium strains that were fed the exchanged diet (control to cadmium or cadmium to control) for one week (Variant 1). After isolation the cells were suspended in PBS and mixed with a H_2_O_2_ solution (treated groups; final concentration 50 μM) or with PBS (reference groups) and incubated for 1 min. Abbreviations: ○ or ■ –mean of medians of fifty nuclei measured on each slide; (C->Cd), (Cd->C)–individuals from the control and cadmium strains that were fed the exchanged diet (control to cadmium or cadmium to control, respectively); 0, 5, 15 or 30 min—time period after the end of the incubation; the same letters indicate homogenous groups within a strain (ANOVA, Tukey test, p < 0.05).

Similar to variant 0, the highest level of DNA damage was found immediately after the addition of H_2_O_2_. The level of DNA damage after the induction by H_2_O_2_ was slightly higher in the C→Cd group compared to the Cd→C group. The level of DNA damage in the reference groups in variant 1 was similar to the values obtained in the basal model (variant 0) (Figs 5 and 6; Table 3; S2 Fig).

**Table 3:** DNA damage (mean ± SD) in the nuclei of the hemocytes of the 5^th^ instar of *S*. *exigua* from the control and cadmium strains that were fed the exchanged diet (C-> Cd: control to cadmium or Cd->C: cadmium to control) for one week (Variant 1). After isolation the cells were suspended in PBS and mixed with a H_2_O_2_ solution (treated groups; final concentration 50 μM) or with PBS (reference groups) and incubated for 1 min.

Parameter	TDNA [%]					TL (μm)					OTM (arbitrary units)			
	C->Cd			Cd->C		C->Cd			Cd->C		C->Cd		Cd->C	
Time interval	M	SD		M	SD	M	SD		M	SD	M	SD	M	SD
*PBS (reference groups)*														
0 min	15.22	4.70		12.80	1.37	8.32	2.74		8.51	1.58	1.58	0.84	1.39	0.43
5 min	13.38	3.40		12.93	2.64	7.48	1.98		6.23	1.56	1.23	0.46	0.97	0.33
15 min	12.34	3.33		11.40	1.52	7.00	1.62	*	5.16	0.72	1.13	0.35	0.91	0.32
30 min	12.32	1.84		11.96	2.66	6.24	0.55		5.96	1.70	1.13	0.22	1.03	0.37
*H*_*2*_*O*_*2*_ *(treated groups)*														
0 min	32.02	5.80		27.39	4.66	17.06	4.88		13.09	1.72	3.86	1.01	3.13	0.41
5 min	24.46	2.61		23.34	3.61	10.92	1.37		10.96	1.29	2.51	0.79	2.20	0.36
15 min	23.18	2.43	*	19.09	0.88	11.26	1.49		9.52	2.18	2.17	0.83	1.64	0.47
30 min	19.23	1.40		17.56	2.39	9.37	1.40		8.68	1.25	1.45	0.52	1.71	0.36

Abbreviations: TDNA—Tail DNA; TL—Tail Length, OTM—Olive Tail Moment; M—mean, SD—standard deviation, *–indicates significant differences between the control and cadmium lines for specific time period (0, 5, 15 or 30 min) at the end of the incubation with H_2_O_2_ (ANOVA, Tukey test, p < 0.05; n = 5 slides with 50 nuclei each in each group).

The level of DNA damage in the reference groups (only PBS) was similar in both strains. The mean TDNA fluctuated between 11.40% and 15.22% (Fig 6; Table 3; PBS treated groups). The highest value of TL in the reference groups was found at time point 0 (S2 Fig, Table 3). No statistically significant differences were found for any of the three parameters of DNA damage in relation to time (Fig 6; S2 Fig).

The induction of DNA damage caused by H_2_O_2_ was always the highest at the first time point. However, unlike in variant 0, the insects from the control strain that were transferred to the Cd-containing food showed a high potential to repair the damage within a short time. Only after a five-minute-long incubation did the TDNA decrease significantly compared to time point 0. However, there were still significant differences between the H_2_O_2_- and PBS-treated groups. This difference only disappeared after the 30-minute-long incubation. At that time point, the level of DNA damage was similar in the H_2_O_2_-treated and reference hemocytes (Fig 6). Hemocytes of the Cd strain that were transferred to the unpolluted diet generally had a lower level of DNA damage compared to the analogous groups in variant 0. Moreover, after 30 minutes of incubation, the differences between the reference and the H_2_O_2-_treated groups were insignificant (Fig 6 –right side of the graphs; S2 Fig).

#### Variant 2 –insects on the exchanged diet for one generation

**Fig 7:**
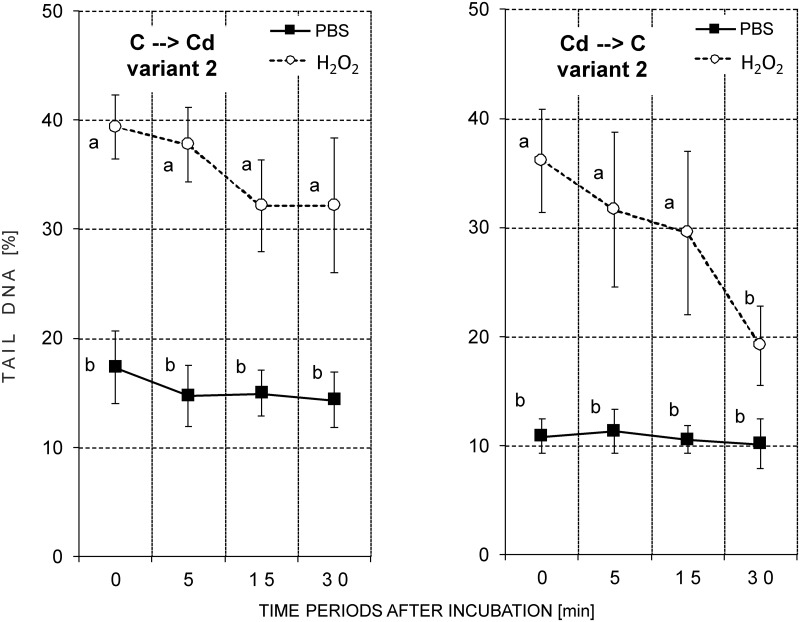
Tail DNA (%; mean ± SD) in the nuclei of the hemocytes of the 5^th^ instar of *S*. *exigua* from the control and cadmium strains that were fed the exchanged diet (control to cadmium or cadmium to control) for one generation (Variant 2). After isolation the cells were suspended in PBS and mixed with a H_2_O_2_ solution (treated groups; final concentration 50 μM) or with PBS (reference groups) and incubated for 1 min. Abbreviations: ○ or ■ –mean of medians of fifty nuclei measured on each slide; (C->Cd), (Cd->C)–individuals from the control and cadmium strains that were fed the exchanged diet (control to cadmium or cadmium to control, respectively); 0, 5, 15 or 30 min—time period after the end of the incubation; the same letters indicate homogenous groups within a strain (ANOVA, Tukey test, p < 0.05).

Compared to the previous two variants, the cells with the highest level of DNA damage were detected in variant 2. It was especially distinct in the case of the insects from the control strain that were fed the Cd-containing diet (Figs 5 and 7; Table 4; S3 Fig).

**Table 4:** DNA damage (mean ± SD) in the nuclei of the hemocytes of the 5^th^ instar of *S*. *exigua* from the control and cadmium strains that were fed the exchanged diet (C-> Cd: control to cadmium or Cd->C: cadmium to control) for one generation (Variant 2). After isolation the cells were suspended in PBS and mixed with a H_2_O_2_ solution (treated groups; final concentration 50 μM) or with PBS (reference groups) and incubated for 1 min.

Parameter	TDNA [%]					TL (μm)				OTM (arbitrary units)			
	C->Cd			Cd->C		C->Cd		Cd->C		C->Cd		Cd->C	
Time interval	M	SD		M	SD	M	SD	M	SD	M	SD	M	SD
*PBS (reference groups)*													
0 min	17.32	3.35	*	10.86	1.56	6.70	2.49	4.06	1.49	1.17	0.49	0.74	0.25
5 min	14.72	2.78		11.32	1.98	4.48	1.66	4.42	1.88	0.84	0.23	0.74	0.20
15 min	14.96	2.13		10.53	1.27	5.04	1.86	5.29	2.33	0.86	0.19	0.67	0.23
30 min	14.33	2.55		10.17	2.31	5.35	1.40	4.12	0.92	0.86	0.10	0.57	0.11
*H*_*2*_*O*_*2*_ *(treated groups)*													
0 min	39.34	2.92		36.12	4.71	16.51	3.31	16.32	2.96	4.53	0.54	4.47	0.96
5 min	37.72	3.42		31.61	7.10	15.52	3.27	16.11	2.56	4.21	1.04	3.75	0.79
15 min	32.14	4.20		29.53	7.49	14.23	2.11	13.68	5.58	3.30	0.99	3.03	1.53
30 min	32.15	6.18	*	19.16	3.65	13.93	1.83	7.45	2.69	3.01	1.14	1.72	0.87

Abbreviations: TDNA—Tail DNA; TL—Tail Length, OTM—Olive Tail Moment; M—mean, SD—standard deviation, *—indicates significant differences between the control and cadmium lines for specific time period (0, 5, 15 or 30 min) at the end of the incubation with H_2_O_2_ (ANOVA, Tukey test, p < 0.05; n = 5 slides with 50 nuclei each in each group).

The level of DNA damage in the reference groups was similar to the values found in variants 0 and 1. The only significant difference between C→Cd and Cd→C appeared at the beginning of the experiment at the time point 0 when the TDNA value in the reference group for the C→Cd strain (mean value: 17.32%) was significantly higher than in the reference group for the Cd→C strain (mean value: 10.86%) (Table 4 –asterisks in the PBS-treated groups). No statistically significant time-dependent differences in the level of DNA damage were found (Fig 7; S3 Fig).

Mean values for TDNA in the hemocytes of the insects from the C→Cd group were between 32.14% and 39.34% (Table 4 –reference groups). No significant time-dependent decrease in this parameter value was found in this strain (Fig 7 –groups marked with circles: ○). In the Cd→C strain, in turn, a gradual decrease in the level of DNA damage was observed over time. Thirty minutes after the beginning of the incubation of cells with H_2_O_2_ or PBS, no statistically significant differences were found between induced and reference groups (Fig 7; S3 Fig).

### AFLP polymorphism

The AFLP analysis of the *S*. *exigua* control and cadmium breeding strains, which was based on 12 primer combinations, resulted in the amplification of 431 loci (S1 Table). Only five of them were polymorphic, which gave the proportion of 1.16% polymorphic loci across all of the samples in the study. The level of polymorphism within each strain was comparable (Table 5). Three individuals from the control strain were represented by the same AFLP fingerprint and, similarly, an identical band pattern was observed for two individuals from the cadmium-bred strain. When comparing these two strains, the same AFLP profiles were found for one individual from the control and for two individuals from the cadmium-bred strain. Importantly, no gain or loss of any of AFLP locus was observed in the cadmium-bred strain. All of the polymorphisms within that strain resulted from the segregation of the bands in these loci, which were also polymorphic within the control strain.

**Table 5:** AFLP polymorphism within and between the *S*. *exigua* control and cadmium-bred strains.

Line	Total No. of loci	No. of polymorphic loci	Proportion of polymorphic loci [%]	Pairwise polymorphism [%]	
				Range	Mean value
**Polymorphism within a line**					
Control	431	5	1.16	0–1.16	0.558
Cadmium	431	5	1.16	0–1.16	0.697
**Polymorphism between lines**					
Control vs Cadmium	431	5	1.16	0–1.16	0.549

## Discussion

The results obtained in this study provide the opportunity to gain more in-depth knowledge of insect defense reactions to the environmental stress that is caused by humans. In this study, the duration of exposure to the toxic factor appeared to be especially important. Anthropogenic modifications of the environment are always rapid, and that is why the adaptation of organisms (in the strict sense) to these changes can progress with unsatisfying effects [30]. Therefore, comparing the results obtained in the laboratory and in field studies allows the basic mechanisms of a better tolerance to be understood.

There are examples of studies that inarguably prove that exposure of insects to metal ions causes a significant increase in DNA damage. For example, the level of the increase in DNA damage, which is manifested by an increase of TDNA to nearly 20% in the hemocytes of *D*. *melanogaster* larvae that had been bred for 24 hours in a medium containing 1 and 2.5 mM of K_2_Cr_2_O_7_, was observed [31]. In other experiments, a significant increase in DNA damage in the hemocytes of *D*. *melanogaster* was observed after a 24-hour exposure to 5–20 mM of NiSO_4_ in [32] or to 2–8 mM of Pb(NO_3_)_2_ [33]. The examples mentioned above, however, concern a short-term (measured in hours) exposure to metals or metal-containing nanoparticles. In these cases, the response to stress, prevention against harmful effects or damage repair is realized using the available enzymatic pool.

In other our research the basic level of DNA damage in the ganglia cells of the brains of newly-hatched grasshopper larvae was positively correlated with the level of contamination of their habitat. Differences were confirmed despite a diapause (12 weeks at 4°C) that was arranged under the same laboratory conditions in unpolluted sand. This means that from the moment that eggs are laid until they hatched, the embryos were subjected to similar conditions. Such a result can be evidence of permanent changes that are possibly consolidated by natural selection [34]. The results described above are in accordance with the results obtained in the present studies for a Cd-exposed strain. These insects had a lower percentage of apoptotic cells (Figs 1 and 2) and their potential to repair H_2_O_2_-caused DNA damage was higher, despite their transfer to the control food in variants 1 and 2 (Figs 4–7). These features were not observed in the control insects.

Our investigations of polymorphism using AFLP did not reveal any differences between the two main insect strains (Table 5). Such a finding suggests that cadmium in the diet of *S*. *exigua* did not induce any novel mutations and that the polymorphisms in the cadmium-bred strain most probably reflect the diversity of the founder population. Moreover, the very low level of variation between the individuals in this analysis shows that both strains are relatively uniform at the genetic level. It is important to remember, however, that AFLP permits only a portion of genome to be analyzed. Therefore, we cannot exclude the fact that the use of other, high-resolution techniques that are based, for example, on next-generation sequencing, would reveal some differences. When looking at the AFLP results together with the results of the comet assays, we may suppose that the insects from the Cd-exposed strain use other forms of nongenetic inheritance in addition to the phenotypic plasticity that is probably responsible for the changes in the case of the short-term Cd exposure in the case of the control strain (Fig 6 variant 1 –strain: C->Cd; S2 Fig). As it is known, nongenetic inheritance involve a range of mechanisms by which the parents can influence the phenotypes of their offspring. Among various mechanisms most frequently mentioned, and worth attention are the transmission of epigenetic variation (DNA-methylation pattern, the chromatin structure or RNA), quality and quantity of nutrients stored in eggs, hormones or enzymes [35]. Cadmium chronically presented in food can stimulate within-generation plasticity (including developmental and phenotypic plasticity), but we can also expect of transgenerational effects. According to our observations, the survivorship of each instar and the lifespan of the insects from both strains is similar. The number of eggs and hatching success is slightly higher in Cd-exposed strain. Preliminary investigations revealed also the differences in the expression and sequence of the vitellogenin gene. These results are prepared for publication.

A short-term (one week) exposure to cadmium possibly leads to the initiation of defense mechanisms (the synthesis of enzymatic proteins) at the cost of the energy reserves of an individual. DNA repair mechanisms have to be active at all times in order to fulfill their role but this is energy consuming. The correct action of these mechanisms has a significant influence on the basic functions of a cell and an organism [36]. For animals that are under the constant pressure of toxic factors (not only genotoxicological ones), damage repair as well as the synthesis of new molecules to replace damaged ones is a serious challenge that requires a compromise between survival and reproduction [37–39]. In this perspective, the successful elimination of DNA damage, despite this being a priority of an organism, may not always be realized at the highest efficiency. When an organism has a short lifespan, such as is the case of many insect species, their main aim can be to invest energy into reproduction at the expense of the quality and length of a parent’s life.

In the case of prolonged intoxication (one generation), this mechanism does not seem to be effective and the defensive response fails (Fig 7, variant 2 –control strain: C->Cd; S3 Fig). A failure like this also leads to a significant decrease in H_2_O_2_ removal from the cells (Fig 3 –variant 2, strain C->Cd), which can be interpreted as a symptom of unfavorable changes in the organism, since it is known that H_2_O_2_ at an optimal concentration takes part in intracellular signaling and regulation. For example, an increase in H_2_O_2_ concentration and a decrease in catalase activity were found in the tissues of *Ostrinia nubilaris* (Lepidoptera: Pyralidae) that were kept at -3^°^C. The authors suggested that H_2_O_2_ may be connected with the regulation of the response to stress and the tolerance to freezing [27].

In our opinion, in the case of the cadmium strain, epigenetic modifications may also take place in addition to the phenotypic plasticity [11]. However, the impact of various non-genetic maternal factors that are transferred during oogenesis on embryonic DNA integrity cannot be ruled out [29]. There are several scientific groups in the world, studying Lepidoptera. The studies, however, have always concerned the assessment of the radiation effects on DNA stability [40–44]. Significant data on the long-term metal exposure on DNA stability in insects can be found in the studies on grasshoppers. *Chorthippus brunneus* larvae that went through an obligatory diapause (12 weeks, 4°C) in soil that had been contaminated with Zn (100 μg Zn·g^−1^ or 1000 μg Zn·g^−1^) had a significantly higher level of damage in the ganglia cells of the brain, but only when the diapause occurred in sand that had a lower metal concentration. It is worth noting that for a higher zinc concentration, both the amount of metal in the brain and the level of DNA damage remained at the control level. It cannot be excluded that above a certain threshold level some protective mechanisms against metal toxicity were activated. Redistribution of xenobiotic and/or immobilization in other body parts may also be factors [34].

Therefore, metal toxicity, which in this case was expressed as the level of DNA damage in insect cells, not only indisputably depends on the dose, but also on the duration of the contact of the animal or population with a certain xenobiotic in the past (or substances with a similar mechanism of action). In this research, we concluded that the basic level of DNA damage in insects from both strains was similar in almost all of the cases (Tables 1, 3 and 4; see reference groups). This means that prolonged contact with cadmium through food supposedly leads to a selection of individuals that are equipped with efficient defense mechanisms. These can include multilevel adaptations ranging from a limited intake of the xenobiotic through the capacity to immobilize it within a cell to improvements in the enzymatic intermediate (e.g. free radicals) elimination systems and the repair of the DNA damage that is caused. From our earlier research on a selected *S*. *exigua* strain, it would seem these processes can occur simultaneously, but at varying intensities [7,8,45,46].

Even though the basic level of DNA damage in *S*. *exigua* larvae from both strains was similar, differences between the selected insect strains appeared after the artificial induction of the damage by the administration of H_2_O_2_ (Table 1 –H_2_O_2_ treated groups). After the induction of DNA strand breaks in the cells of the insects from the cadmium strain, damage levels decreased gradually and reached values close to the reference values (cell suspension treated with PBS instead of H_2_O_2_) after 30 minutes (Fig 4 –see cadmium strain). The time it took for the DNA breaks to rejoin after the H_2_O_2_-induced damage was rapid and similar to other cells. For most cells, the time was short with a half-time of a few minutes [16,47]. For example, the repair time for U937 was about 20 min [48]. In this study the slightly higher level of DNA damage that was observed in the reference groups at time point 0 was statistically insignificant compared to the remaining time points. This may be connected with the induction of additional DNA damage () after the acute exposure of the hemocytes to atmospheric oxygen immediately after releasing the hemolymph from the insect body as was suggested in the case of lymphocytes [47].

One more question arises: How does a cell (and an organism) cope when the concentration of a stress agent is extremely low? One explanation might come from the results obtained in this research for the control strain of *S*. *exigua* (variant 0). These insects had not been exposed to any stressing factors for many generations and no symptoms of repair of DNA damage that was induced by H_2_O_2_ within 30 minutes were observed (Fig 4 –see control strain; S1 Fig). We find this result very interesting and difficult to explain. Surprisingly, the insects from the control strain were more sensitive and did not cope very well after the application of a DNA-damaging agent.

The results of our experiment on selected strains seem to be in agreement with the idea of stress, which assumes that moderate stress is necessary in order to maintain the proper fitness and development of an animal. Moderate stress keeps an organism in a state of readiness and mobilization [49]. At the population level, stress is an important factor in species evolution (especially in a changing environment). Therefore, moderate stress (also anthropogenic) plays an important role in the development of tolerance to local conditions, thus helping in the better adjustment, synchronization and functioning of organisms [49].

The prolonged selection of *S*. *exigua* for cadmium resistance in our laboratory simulated the placement of animals outside of their ecological niche, thus likely inducing changes in the bodies of the animals (on the physiological and biochemical levels). These insects are therefore unique material for the further study of the environmental stress response and adaptation mechanisms.

To summarize, the high potential for DNA damage repair that was found in the insects from the cadmium strain may confirm the (ii) hypothesis, which suggests that multigenerational exposure to that metal may possibly contribute to the selection of insects that have a wider tolerance to oxidative stress.

## Supporting Information

S1 Fig Tail length and olive tail moment in *S*. *exigua* hemocyte nuclei (Variant 0). (PDF)

S2 Fig Tail length and olive tail moment in *S*. *exigua hemocyte* nuclei (Variant 1). (PDF)

S3 Fig Tail length and olive tail moment in *S*. *exigua* hemocyte nuclei (Variant 2). (PDF)

S1 Table The matrix of AFLP data generated after the analysis of 12 primer combinations for *S*. *exigua* control and cadmium breeding strains. (PDF)
