# Supplementary material for: Phenotypic Plasticity, Epigenetic or Genetic Modifications in Relation to the Duration of Cd-Exposure within a Microevolution Time Range in the Beet Armyworm
Source: PLoS One. 2016 Dec 1;11(12):e0167371. doi: 10.1371/journal.pone.0167371 (PMC5131940; doi:10.1371/journal.pone.0167371)

**S1 Fig. Tail length (a) and olive tail moment (b) in *S. exigua* hemocyte nuclei (Variant 0).**

(a) Tail length (µm; mean ± SD) in the nuclei of the hemocytes of the 5th instar of *S. exigua* from the control and cadmium strains (Variant 0). After isolation the cells were suspended in PBS and mixed with an $H_2O_2$ solution (treated groups; final concentration 50 µM) or with PBS (reference groups) and incubated for 1 min.

Abbreviations: ○ or ■ – mean of the medians of fifty nuclei that were measured on each slide; 0, 5, 15 or 30 min – time period after the end of the incubation; the same letters indicate homogenous groups within a strain (ANOVA, Tukey test, $p < 0.05$).

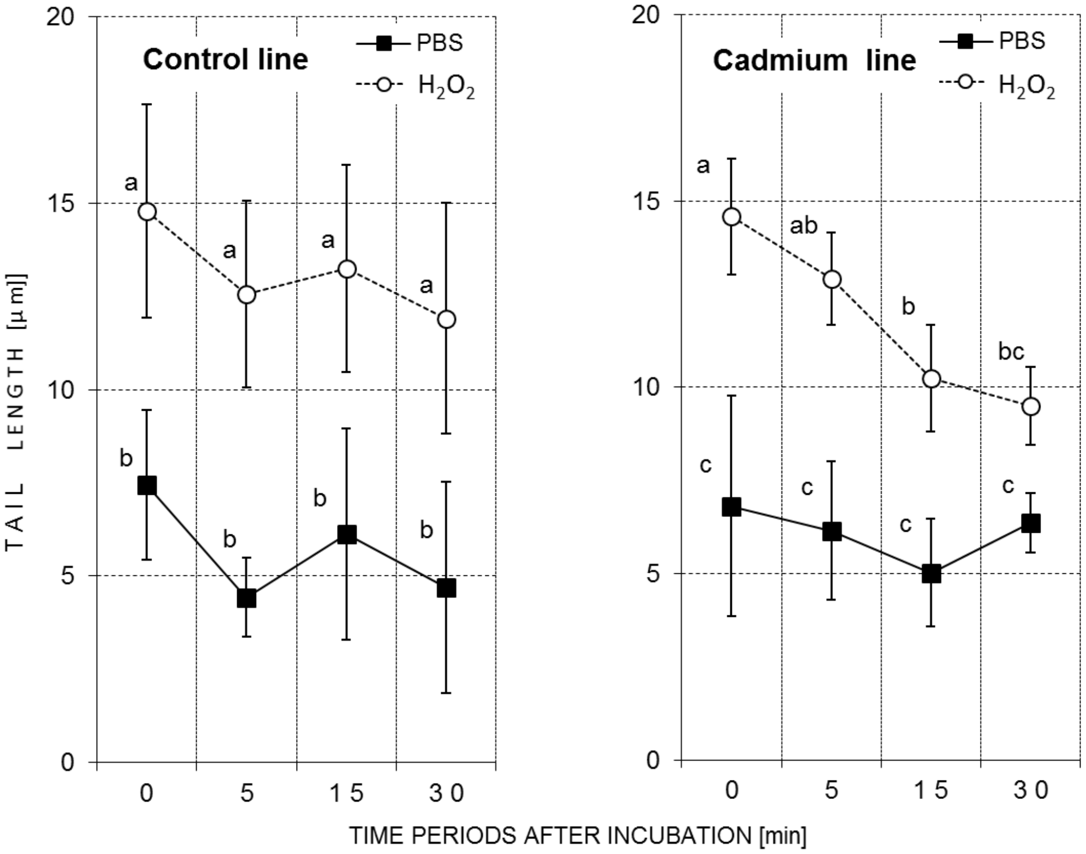

(b) Olive tail moment (arbitrary units; mean ± SD) in the nuclei of the hemocytes of the 5th instar of *S. exigua* from the control and cadmium strains (Variant 0). After isolation the cells were suspended in PBS and mixed with an $H_2O_2$ solution (treated groups; final concentration 50 µM) or with PBS (reference groups) and incubated for 1 min.

Abbreviations: ○ or ■ – mean of the medians of fifty nuclei that were measured on each slide; 0, 5, 15 or 30 min – time period after the end of the incubation; the same letters indicate homogenous groups within a strain (ANOVA, Tukey test, $p < 0.05$).

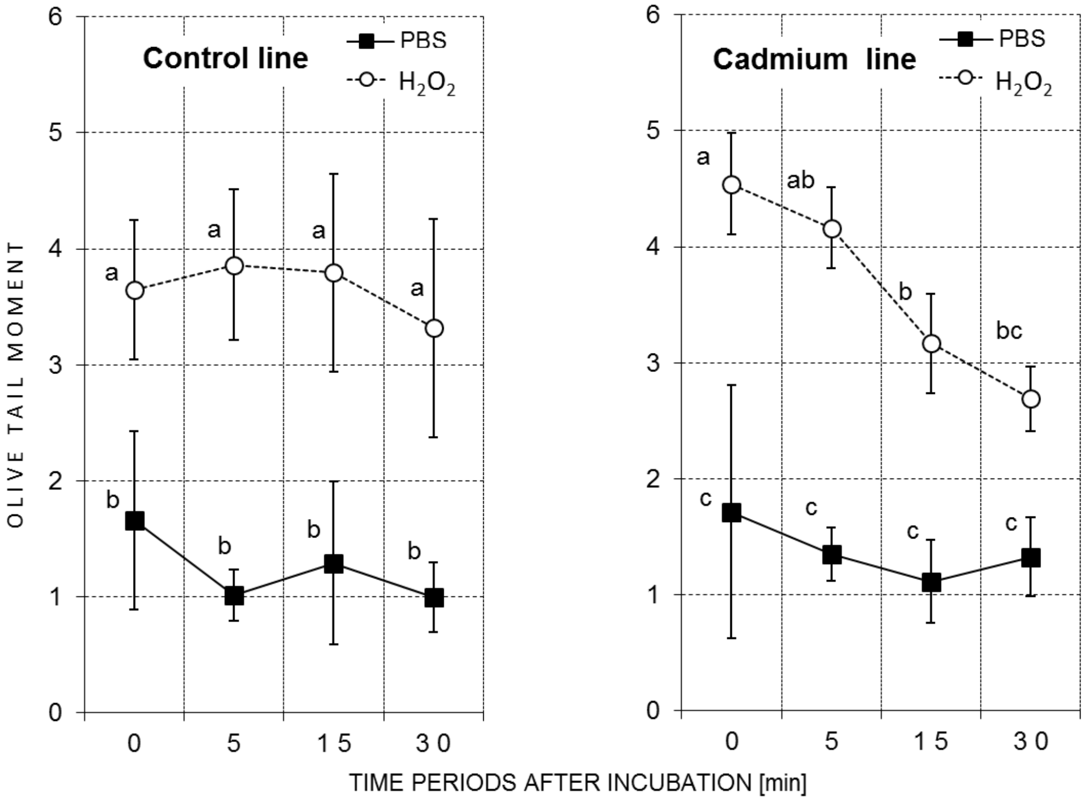

Supplement: S1 Fig — (PDF) [file pone.0167371.s001.pdf]
