# Supplementary material for: Phenotypic Plasticity, Epigenetic or Genetic Modifications in Relation to the Duration of Cd-Exposure within a Microevolution Time Range in the Beet Armyworm
Source: PLoS One. 2016 Dec 1;11(12):e0167371. doi: 10.1371/journal.pone.0167371 (PMC5131940; doi:10.1371/journal.pone.0167371)

S3 Fig. Tail length (a) and olive tail moment (b) in *S. exigua* hemocyte nuclei (Variant 2).

(a) Tail length (µm; mean ± SD) in the nuclei of the hemocytes of the 5th instar of *S. exigua* from the control and cadmium strains that were fed the exchanged diet (control to cadmium or cadmium to control) throughout 1 generation (Variant 2). After isolation the cells were suspended in PBS and mixed with an H_2O_2 solution (treated groups; final concentration 50 µM) or with PBS (reference groups) and incubated for 1 min.

Abbreviations: ○ or ■ – mean of medians of fifty nuclei measured on each slide; (C->Cd), (Cd->C) – individuals from the control and cadmium strains that were fed the exchanged diet (control to cadmium or cadmium to control, respectively); 0, 5, 15 or 30 min – time period after the end of the incubation; the same letters indicate homogenous groups within a strain (ANOVA, Tukey test, $p < 0.05$).

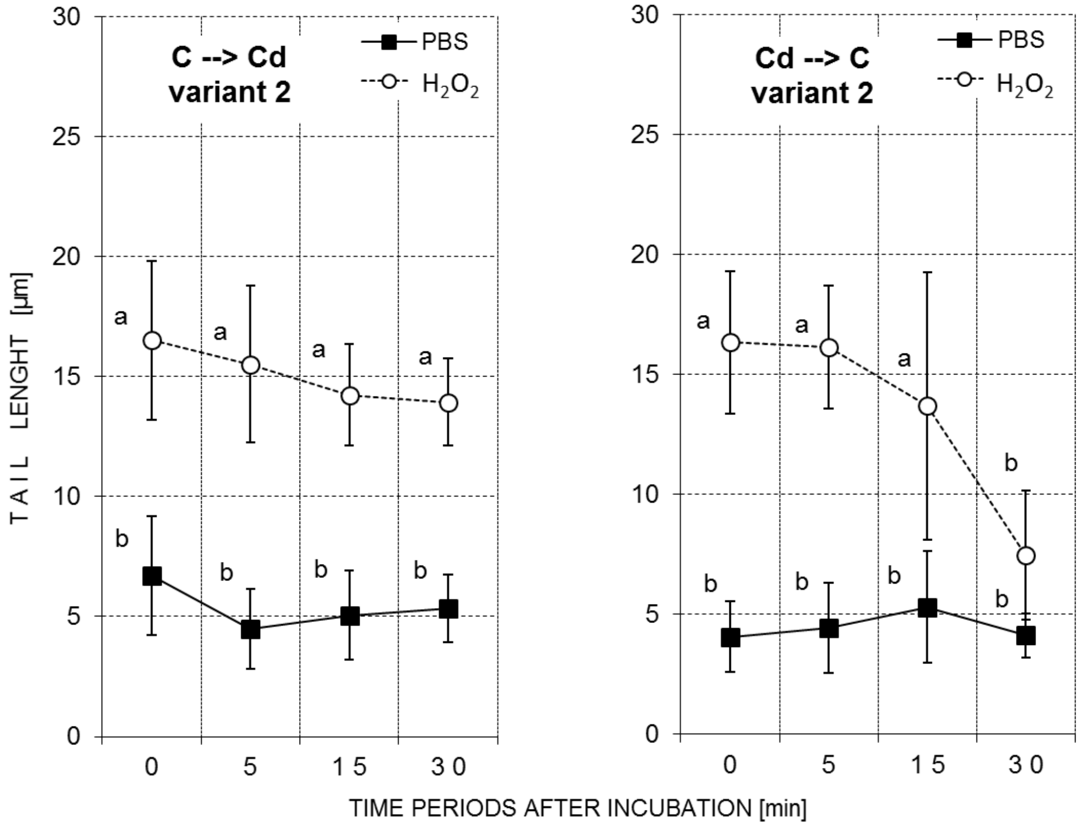

(b) Olive tail moment (in arbitrary units; mean ± SD) in the nuclei of the hemocytes of the 5th instar of *S. exigua* from the control and cadmium strains that were fed the exchanged diet (control to cadmium or cadmium to control) for one generation (Variant 2). After isolation the cells were suspended in PBS and mixed with an H_2O_2 solution (treated groups; final concentration 50 μM) or with PBS (reference groups) and incubated for 1 min.

Abbreviations: ○ or ■ – mean of medians of fifty nuclei measured on each slide; (C->Cd), (Cd->C) – individuals from the control and cadmium strains that were fed the exchanged diet (control to cadmium or cadmium to control, respectively); 0, 5, 15 or 30 min – time period after the end of the incubation; the same letters indicate homogenous groups within a strain (ANOVA, Tukey test, $p < 0.05$).

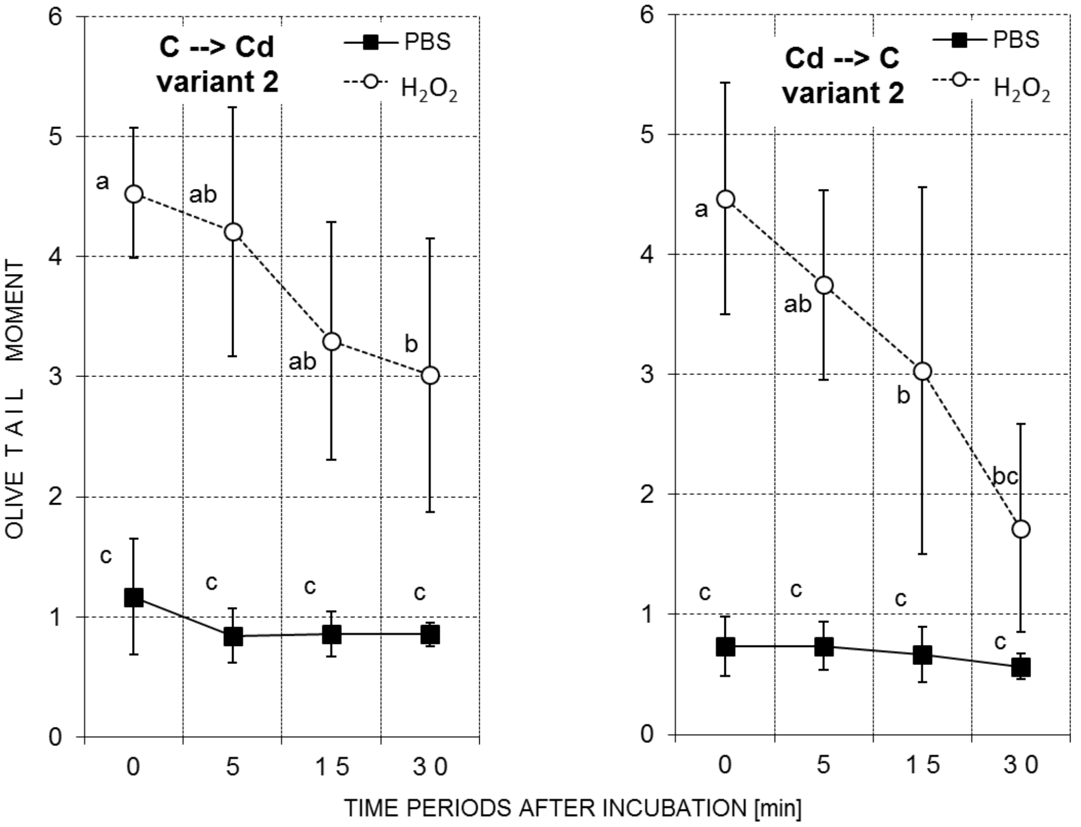

Supplement: S3 Fig — (PDF) [file pone.0167371.s003.pdf]
