# Supplementary material for: Phenotypic Plasticity, Epigenetic or Genetic Modifications in Relation to the Duration of Cd-Exposure within a Microevolution Time Range in the Beet Armyworm
Source: PLoS One. 2016 Dec 1;11(12):e0167371. doi: 10.1371/journal.pone.0167371 (PMC5131940; doi:10.1371/journal.pone.0167371)
Supplement: S1 Table — (PDF) [file pone.0167371.s004.pdf]

S1 Table

The matrix of AFLP data generated after the analysis of 12 primer combinations
for *S. exigua* control and cadmium breeding strains

Legend: C - control strain; Cd - cadmium breeding strain; polymorphic loci are marked in red

Primer combination	Locus No.	Samples codes									
		C_1	C_2	C_3	C_4	C_5	Cd_1	Cd_2	Cd_3	Cd_4	Cd_5
E-AAG/M-CAA	1	1	1	1	1	1	1	1	1	1	1
	2	1	1	1	1	1	1	1	1	1	1
	3	1	1	1	1	1	1	1	1	1	1
	4	1	1	1	1	1	1	1	1	1	1
	5	1	1	1	1	1	1	1	1	1	1
	6	1	1	1	1	1	1	1	1	1	1
	7	1	1	1	1	1	1	1	1	1	1
	8	1	1	1	1	1	1	1	1	1	1
	9	1	1	1	1	1	1	1	1	1	1
	10	1	1	1	1	1	1	1	1	1	1
	11	1	1	1	1	1	1	1	1	1	1
	12	1	1	1	1	1	1	1	1	1	1
	13	1	1	1	1	1	1	1	1	1	1
	14	1	1	1	1	1	1	1	1	1	1
	15	1	1	1	1	1	1	1	1	1	1
	16	1	1	1	1	1	1	1	1	1	1
	17	1	1	1	1	1	1	1	1	1	1
	18	1	1	1	1	1	1	1	1	1	1
	19	1	1	1	1	1	1	1	1	1	1
	20	1	1	1	1	1	1	1	1	1	1
	21	1	1	1	1	1	1	1	1	1	1
	22	1	1	1	1	1	1	1	1	1	1
	23	1	1	1	1	1	1	1	1	1	1
	24	1	1	1	1	1	1	1	1	1	1
	25	1	1	1	1	1	1	1	1	1	1
	26	1	1	1	1	1	1	1	1	1	1
	27	1	1	1	1	1	1	1	1	1	1
	28	1	1	1	1	1	1	1	1	1	1
	29	1	1	1	1	1	1	1	1	1	1
	30	1	1	1	1	1	1	1	1	1	1
	31	1	1	1	1	1	1	1	1	1	1
	32	1	1	1	1	1	1	1	1	1	1
E-AG/M-CAA	1	1	1	1	1	1	1	1	1	1	1
	2	1	1	1	1	1	1	1	1	1	1
	3	1	1	1	1	1	1	1	1	1	1
	4	1	1	1	1	1	1	1	1	1	1
	5	1	1	1	1	1	1	1	1	1	1
	6	1	1	1	1	1	1	1	1	1	1
	7	1	1	1	1	1	1	1	1	1	1
	8	1	1	1	1	1	1	1	1	1	1
	9	1	1	1	1	1	1	1	1	1	1
	10	1	1	1	1	1	1	1	1	1	1

	11	1	1	1	1	1	1	1	1	1	1
	12	1	1	1	1	1	1	1	1	1	1
	13	1	1	1	1	1	1	1	1	1	1
	14	1	1	1	1	1	1	1	1	1	1
	15	1	1	1	1	1	1	1	1	1	1
	16	1	1	1	1	1	1	1	1	1	1
	17	1	1	1	1	1	1	1	1	1	1
	18	1	1	1	1	1	1	1	1	1	1
	19	1	1	1	1	1	1	1	1	1	1
	20	1	1	1	1	1	1	1	1	1	1
	21	1	1	1	1	1	1	1	1	1	1
	22	1	1	1	1	1	1	1	1	1	1
	23	1	1	1	1	1	1	1	1	1	1
	24	1	1	1	1	1	1	1	1	1	1
	25	1	1	1	1	1	1	1	1	1	1
	26	1	1	1	1	1	1	1	1	1	1
	27	1	1	1	1	1	1	1	1	1	1
	28	1	1	1	1	1	1	1	1	1	1
	29	1	1	1	1	1	1	1	1	1	1
	30	1	1	1	1	1	1	1	1	1	1
	31	1	0	1	0	1	0	0	1	0	1
	32	1	1	1	1	1	1	1	1	1	1
	33	1	1	1	1	1	1	1	1	1	1
	34	1	1	1	1	1	1	1	1	1	1
	35	1	1	1	1	1	1	1	1	1	1
	36	1	1	1	1	1	1	1	1	1	1
	37	1	1	1	1	1	1	1	1	1	1
	38	1	1	1	1	1	1	1	1	1	1
	39	1	1	1	1	1	1	1	1	1	1
	40	1	1	1	1	1	1	1	1	1	1
	41	1	1	1	1	1	1	1	1	1	1
	42	1	1	1	1	1	1	1	1	1	1
	43	1	1	1	1	1	1	1	1	1	1
	44	1	1	1	1	1	1	1	1	1	1
	45	1	1	1	1	1	1	1	1	1	1
	46	1	1	1	1	1	1	1	1	1	1
	47	1	1	1	1	1	1	1	1	1	1
	48	1	1	1	1	1	1	1	1	1	1
	49	1	1	1	1	1	1	1	1	1	1
	50	1	1	1	1	1	1	1	1	1	1
	51	1	1	1	1	1	1	1	1	1	1
	52	1	1	1	1	1	1	1	1	1	1
	53	1	1	1	1	1	1	1	1	1	1
	54	1	1	1	1	1	1	1	1	1	1
	55	1	1	1	1	1	1	1	1	1	1
	56	1	1	1	1	1	1	1	1	1	1
	57	1	1	1	1	1	1	1	1	1	1
	58	1	1	1	1	1	1	1	1	1	1
E-AA/M-CAA	1	1	1	1	1	1	1	1	1	1	1
	2	1	1	1	1	1	1	1	1	1	1

	3	1	1	1	1	1	1	1	1	1	1
	4	1	1	1	1	1	1	1	1	1	1
	5	1	1	1	1	1	1	1	1	1	1
	6	1	1	1	1	1	1	1	1	1	1
	7	1	1	1	1	1	1	1	1	1	1
	8	1	1	1	1	1	1	1	1	1	1
	9	1	1	1	1	1	1	1	1	1	1
	10	1	1	1	1	1	1	1	1	1	1
	11	1	1	1	1	1	1	1	1	1	1
	12	1	1	1	1	1	1	1	1	1	1
	13	1	1	1	1	1	1	1	1	1	1
	14	1	1	1	1	1	1	1	1	1	1
	15	1	1	1	1	1	1	1	1	1	1
	16	1	1	1	1	1	1	1	1	1	1
	17	1	1	1	1	1	1	1	1	1	1
	18	1	1	1	1	1	1	1	1	1	1
	19	1	1	1	1	1	1	1	1	1	1
	20	1	1	1	1	1	1	1	1	1	1
	21	1	1	1	1	1	1	1	1	1	1
	22	1	1	1	1	1	1	1	1	1	1
	23	1	1	1	1	1	1	1	1	1	1
	24	1	1	1	1	1	1	1	1	1	1
	25	1	1	1	1	1	1	1	1	1	1
	26	1	1	1	1	1	1	1	1	1	1
	27	1	1	1	1	1	1	1	1	1	1
	28	1	1	1	1	1	1	1	1	1	1
	29	1	1	1	1	1	1	1	1	1	1
	30	1	1	1	1	1	1	1	1	1	1
	31	1	1	1	1	1	1	1	1	1	1
	32	1	1	1	1	1	1	1	1	1	1
	33	1	1	1	1	1	1	1	1	1	1
	34	1	1	1	1	1	1	1	1	1	1
	35	1	1	1	1	1	1	1	1	1	1
	36	1	1	1	1	1	1	1	1	1	1
	37	1	1	1	1	1	1	1	1	1	1
	38	1	1	1	1	1	1	1	1	1	1
	39	1	1	1	1	1	1	1	1	1	1
	40	1	1	1	1	1	1	1	1	1	1
	41	1	1	1	1	1	1	1	1	1	1
	42	1	1	1	1	1	1	1	1	1	1
	43	1	1	1	1	1	1	1	1	1	1
	44	1	1	1	1	1	1	1	1	1	1
	45	1	1	1	1	1	1	1	1	1	1
E-AT/M-CAA	1	1	1	1	1	1	1	1	1	1	1
	2	1	1	1	1	1	1	1	1	1	1
	3	1	1	1	1	1	1	1	1	1	1
	4	1	1	1	1	1	1	1	1	1	1
	5	1	1	1	1	1	1	1	1	1	1
	6	1	1	1	1	1	1	1	1	1	1
	7	1	1	1	1	1	1	1	1	1	1

	8	1	1	1	1	1	1	1	1	1	1
	9	1	1	1	1	1	1	1	1	1	1
	10	1	1	1	1	1	1	1	1	1	1
	11	1	1	1	1	1	1	1	1	1	1
	12	1	1	1	1	1	1	1	1	1	1
	13	1	1	1	1	1	1	1	1	1	1
	14	1	1	1	1	1	1	1	1	1	1
	15	1	1	1	1	1	1	1	1	1	1
	16	1	1	1	1	1	1	1	1	1	1
	17	1	1	1	1	1	1	1	1	1	1
	18	1	1	1	1	1	1	1	1	1	1
	19	1	1	1	1	1	1	1	1	1	1
	20	1	1	1	1	1	1	1	1	1	1
	21	1	1	1	1	1	1	1	1	1	1
	22	1	1	1	1	1	1	1	1	1	1
	23	1	1	1	1	1	1	1	1	1	1
	24	1	1	1	1	1	1	1	1	1	1
	25	1	1	1	1	1	1	1	1	1	1
	26	1	1	1	1	1	1	1	1	1	1
	27	1	1	1	1	1	1	1	1	1	1
	28	1	1	1	1	1	1	1	1	1	1
	29	1	1	1	1	1	1	1	1	1	1
	30	1	1	1	1	1	1	1	1	1	1
	31	1	1	1	1	1	1	1	1	1	1
	32	1	1	1	1	1	1	1	1	1	1
	33	1	1	1	1	1	1	1	1	1	1
	34	1	1	1	1	1	1	1	1	1	1
	35	1	1	1	1	1	1	1	1	1	1
	36	1	1	1	1	1	1	1	1	1	1
	37	1	1	1	1	1	1	1	1	1	1
	38	1	1	1	1	1	1	1	1	1	1
	39	1	1	1	1	1	1	1	1	1	1
	40	1	1	1	1	1	1	1	1	1	1
	41	1	1	1	1	1	1	1	1	1	1
	42	1	1	1	1	1	1	1	1	1	1
	43	1	1	1	1	1	1	1	1	1	1
	44	1	1	1	1	1	1	1	1	1	1
E-ACC/M-CAA	1	1	1	1	1	1	1	1	1	1	1
	2	1	1	1	1	1	1	1	1	1	1
	3	1	0	1	0	1	0	0	1	0	1
	4	1	1	1	1	1	1	1	1	1	1
	5	1	1	1	1	1	1	1	1	1	1
	6	1	1	1	1	1	1	1	1	1	1
	7	1	1	1	1	1	1	1	1	1	1
	8	1	1	1	1	1	1	1	1	1	1
	9	1	1	1	1	1	1	1	1	1	1
	10	1	1	1	1	1	1	1	1	1	1
	11	1	1	1	1	1	1	1	1	1	1
	12	1	1	1	1	1	1	1	1	1	1
	13	1	1	1	1	1	1	1	1	1	1

	14	1	1	1	1	1	1	1	1	1	1
	15	1	1	1	1	1	1	1	1	1	1
	16	1	1	1	1	1	1	1	1	1	1
	17	1	1	1	1	1	1	1	1	1	1
	18	1	1	1	1	1	1	1	1	1	1
	19	1	1	1	1	1	1	1	1	1	1
	20	1	1	1	1	1	1	1	1	1	1
	21	1	1	1	1	1	1	1	1	1	1
	22	1	1	1	0	1	1	1	0	0	1
	23	1	1	1	1	1	1	1	1	1	1
	24	1	1	1	1	1	1	1	1	1	1
E-ACC/M-CAG	1	1	1	1	1	1	1	1	1	1	1
	2	1	1	1	1	1	1	1	1	1	1
	3	1	1	1	1	1	1	1	1	1	1
	4	1	1	1	1	1	1	1	1	1	1
	5	1	1	1	1	1	1	1	1	1	1
	6	1	1	1	1	1	1	1	1	1	1
	7	1	1	1	1	1	1	1	1	1	1
	8	1	1	1	1	1	1	1	1	1	1
	9	1	1	1	1	1	1	1	1	1	1
	10	1	1	1	1	1	1	1	1	1	1
	11	1	1	1	1	1	1	1	1	1	1
	12	1	1	1	1	1	1	1	1	1	1
	13	1	1	1	1	1	1	1	1	1	1
	14	1	1	1	1	1	1	1	1	1	1
	15	1	1	1	1	1	1	1	1	1	1
	16	1	1	1	1	1	1	1	1	1	1
	17	1	1	1	1	1	1	1	1	1	1
	18	1	1	1	1	1	1	1	1	1	1
	19	1	1	1	1	1	1	1	1	1	1
	20	1	1	1	1	1	1	1	1	1	1
	21	1	1	1	1	1	1	1	1	1	1
	22	1	1	1	1	1	1	1	1	1	1
	23	1	1	1	1	1	1	1	1	1	1
	24	1	1	1	1	1	1	1	1	1	1
	25	1	1	1	1	1	1	1	1	1	1
	26	1	1	1	1	1	1	1	1	1	1
E-ACC/M-CTA	1	1	1	1	1	1	1	1	1	1	1
	2	1	1	1	1	1	1	1	1	1	1
	3	1	1	1	1	1	1	1	1	1	1
	4	1	1	1	1	1	1	1	1	1	1
	5	1	1	1	1	1	1	1	1	1	1
	6	1	1	1	1	1	1	1	1	1	1
	7	1	1	1	1	1	1	1	1	1	1
	8	1	1	1	1	1	1	1	1	1	1
	9	1	1	1	1	1	1	1	1	1	1
	10	1	1	1	1	1	1	1	1	1	1
	11	1	1	1	1	1	1	1	1	1	1
	12	1	1	1	1	1	1	1	1	1	1
	13	1	1	1	1	1	1	1	1	1	1

	14	1	1	1	1	1	1	1	1	1	1
	15	1	1	1	1	1	1	1	1	1	1
	16	1	1	1	1	1	1	1	1	1	1
	17	1	1	1	1	1	1	1	1	1	1
	18	1	1	1	1	1	1	1	1	1	1
	19	1	1	1	1	1	1	1	1	1	1
	20	1	1	1	1	1	1	1	1	1	1
E-ACC/M-CAT	1	1	1	1	1	1	1	1	1	1	1
	2	1	1	1	1	1	1	1	1	1	1
	3	1	1	1	1	1	1	1	1	1	1
	4	1	1	1	1	1	1	1	1	1	1
	5	1	1	1	1	1	1	1	1	1	1
	6	1	1	1	1	1	1	1	1	1	1
	7	1	1	1	1	1	1	1	1	1	1
	8	1	1	1	1	1	1	1	1	1	1
	9	1	1	1	1	1	1	1	1	1	1
	10	1	1	1	1	1	1	1	1	1	1
	11	1	1	1	1	1	1	1	1	1	1
	12	1	1	1	1	1	1	1	1	1	1
	13	1	1	1	1	1	1	1	1	1	1
	14	1	1	1	1	1	1	1	1	1	1
	15	1	1	1	1	1	1	1	1	1	1
	16	1	1	1	1	1	1	1	1	1	1
	17	1	1	1	1	1	1	1	1	1	1
	18	1	1	1	1	1	1	1	1	1	1
	19	1	1	1	1	1	1	1	1	1	1
	20	1	1	1	1	1	1	1	1	1	1
	21	1	1	1	1	1	1	1	1	1	1
	22	1	1	1	1	1	1	1	1	1	1
	23	1	1	1	1	1	1	1	1	1	1
	24	1	1	1	1	1	1	1	1	1	1
	25	1	1	1	1	1	1	1	1	1	1
	26	1	1	1	1	1	1	1	1	1	1
	27	1	1	1	1	1	1	1	1	1	1
	28	1	1	1	1	1	1	1	1	1	1
	29	1	1	1	1	1	1	1	1	1	1
	30	1	1	1	1	1	1	1	1	1	1
	31	1	1	1	1	1	1	1	1	1	1
	32	1	1	1	1	1	1	1	1	1	1
	33	1	1	1	1	1	1	1	1	1	1
	34	1	1	1	1	1	1	1	1	1	1
	35	1	1	1	1	1	1	1	1	1	1
E-ACC/M-CAC	1	1	1	1	1	1	1	1	1	1	1
	2	1	1	1	1	1	1	1	1	1	1
	3	1	1	1	1	1	1	1	1	1	1
	4	1	1	1	1	1	1	1	1	1	1
	5	1	1	1	1	1	1	1	1	1	1
	6	1	1	1	1	1	1	1	1	1	1
	7	1	1	1	1	1	1	1	1	1	1
	8	1	1	1	1	1	1	1	1	1	1

	9	1	1	1	1	1	1	1	1	1	1
	10	1	1	1	1	1	1	1	1	1	1
	11	1	1	1	1	1	1	1	1	1	1
	12	1	1	1	1	1	1	1	1	1	1
	13	1	1	1	1	1	1	1	1	1	1
	14	1	1	1	1	1	1	1	1	1	1
	15	1	1	1	1	1	1	1	1	1	1
	16	1	1	1	1	1	1	1	1	1	1
	17	1	1	1	1	1	1	1	1	1	1
	18	1	1	1	1	1	1	1	1	1	1
	19	0	0	0	1	0	0	0	1	0	1
	20	0	0	0	1	0	0	0	1	0	1
	21	1	1	1	1	1	1	1	1	1	1
	22	1	1	1	1	1	1	1	1	1	1
	23	1	1	1	1	1	1	1	1	1	1
	24	1	1	1	1	1	1	1	1	1	1
	25	1	1	1	1	1	1	1	1	1	1
	26	1	1	1	1	1	1	1	1	1	1
	27	1	1	1	1	1	1	1	1	1	1
	28	1	1	1	1	1	1	1	1	1	1
	29	1	1	1	1	1	1	1	1	1	1
	30	1	1	1	1	1	1	1	1	1	1
	31	1	1	1	1	1	1	1	1	1	1
	32	1	1	1	1	1	1	1	1	1	1
E-AC/M-CAC	1	1	1	1	1	1	1	1	1	1	1
	2	1	1	1	1	1	1	1	1	1	1
	3	1	1	1	1	1	1	1	1	1	1
	4	1	1	1	1	1	1	1	1	1	1
	5	1	1	1	1	1	1	1	1	1	1
	6	1	1	1	1	1	1	1	1	1	1
	7	1	1	1	1	1	1	1	1	1	1
	8	1	1	1	1	1	1	1	1	1	1
	9	1	1	1	1	1	1	1	1	1	1
	10	1	1	1	1	1	1	1	1	1	1
	11	1	1	1	1	1	1	1	1	1	1
	12	1	1	1	1	1	1	1	1	1	1
	13	1	1	1	1	1	1	1	1	1	1
	14	1	1	1	1	1	1	1	1	1	1
	15	1	1	1	1	1	1	1	1	1	1
	16	1	1	1	1	1	1	1	1	1	1
	17	1	1	1	1	1	1	1	1	1	1
	18	1	1	1	1	1	1	1	1	1	1
	19	1	1	1	1	1	1	1	1	1	1
	20	1	1	1	1	1	1	1	1	1	1
	21	1	1	1	1	1	1	1	1	1	1
	22	1	1	1	1	1	1	1	1	1	1
	23	1	1	1	1	1	1	1	1	1	1
	24	1	1	1	1	1	1	1	1	1	1
	25	1	1	1	1	1	1	1	1	1	1
	26	1	1	1	1	1	1	1	1	1	1

	27	1	1	1	1	1	1	1	1	1	1
	28	1	1	1	1	1	1	1	1	1	1
	29	1	1	1	1	1	1	1	1	1	1
	30	1	1	1	1	1	1	1	1	1	1
	31	1	1	1	1	1	1	1	1	1	1
	32	1	1	1	1	1	1	1	1	1	1
	33	1	1	1	1	1	1	1	1	1	1
	34	1	1	1	1	1	1	1	1	1	1
	35	1	1	1	1	1	1	1	1	1	1
	36	1	1	1	1	1	1	1	1	1	1
	37	1	1	1	1	1	1	1	1	1	1
	38	1	1	1	1	1	1	1	1	1	1
	39	1	1	1	1	1	1	1	1	1	1
	40	1	1	1	1	1	1	1	1	1	1
	41	1	1	1	1	1	1	1	1	1	1
	42	1	1	1	1	1	1	1	1	1	1
	43	1	1	1	1	1	1	1	1	1	1
E-AC/M-CAG	1	1	1	1	1	1	1	1	1	1	1
	2	1	1	1	1	1	1	1	1	1	1
	3	1	1	1	1	1	1	1	1	1	1
	4	1	1	1	1	1	1	1	1	1	1
	5	1	1	1	1	1	1	1	1	1	1
	6	1	1	1	1	1	1	1	1	1	1
	7	1	1	1	1	1	1	1	1	1	1
	8	1	1	1	1	1	1	1	1	1	1
	9	1	1	1	1	1	1	1	1	1	1
	10	1	1	1	1	1	1	1	1	1	1
	11	1	1	1	1	1	1	1	1	1	1
	12	1	1	1	1	1	1	1	1	1	1
	13	1	1	1	1	1	1	1	1	1	1
	14	1	1	1	1	1	1	1	1	1	1
	15	1	1	1	1	1	1	1	1	1	1
	16	1	1	1	1	1	1	1	1	1	1
	17	1	1	1	1	1	1	1	1	1	1
	18	1	1	1	1	1	1	1	1	1	1
	19	1	1	1	1	1	1	1	1	1	1
	20	1	1	1	1	1	1	1	1	1	1
	21	1	1	1	1	1	1	1	1	1	1
	22	1	1	1	1	1	1	1	1	1	1
	23	1	1	1	1	1	1	1	1	1	1
	24	1	1	1	1	1	1	1	1	1	1
	25	1	1	1	1	1	1	1	1	1	1
	26	1	1	1	1	1	1	1	1	1	1
	27	1	1	1	1	1	1	1	1	1	1
	28	1	1	1	1	1	1	1	1	1	1
	29	1	1	1	1	1	1	1	1	1	1
	30	1	1	1	1	1	1	1	1	1	1
	31	1	1	1	1	1	1	1	1	1	1
	32	1	1	1	1	1	1	1	1	1	1
	33	1	1	1	1	1	1	1	1	1	1

	34	1	1	1	1	1	1	1	1	1	1
	35	1	1	1	1	1	1	1	1	1	1
	36	1	1	1	1	1	1	1	1	1	1
E-AC/M-CTA	1	1	1	1	1	1	1	1	1	1	1
	2	1	1	1	1	1	1	1	1	1	1
	3	1	1	1	1	1	1	1	1	1	1
	4	1	1	1	1	1	1	1	1	1	1
	5	1	1	1	1	1	1	1	1	1	1
	6	1	1	1	1	1	1	1	1	1	1
	7	1	1	1	1	1	1	1	1	1	1
	8	1	1	1	1	1	1	1	1	1	1
	9	1	1	1	1	1	1	1	1	1	1
	10	1	1	1	1	1	1	1	1	1	1
	11	1	1	1	1	1	1	1	1	1	1
	12	1	1	1	1	1	1	1	1	1	1
	13	1	1	1	1	1	1	1	1	1	1
	14	1	1	1	1	1	1	1	1	1	1
	15	1	1	1	1	1	1	1	1	1	1
	16	1	1	1	1	1	1	1	1	1	1
	17	1	1	1	1	1	1	1	1	1	1
	18	1	1	1	1	1	1	1	1	1	1
	19	1	1	1	1	1	1	1	1	1	1
	20	1	1	1	1	1	1	1	1	1	1
	21	1	1	1	1	1	1	1	1	1	1
	22	1	1	1	1	1	1	1	1	1	1
	23	1	1	1	1	1	1	1	1	1	1
	24	1	1	1	1	1	1	1	1	1	1
	25	1	1	1	1	1	1	1	1	1	1
	26	1	1	1	1	1	1	1	1	1	1
	27	1	1	1	1	1	1	1	1	1	1
	28	1	1	1	1	1	1	1	1	1	1
	29	1	1	1	1	1	1	1	1	1	1
	30	1	1	1	1	1	1	1	1	1	1
	31	1	1	1	1	1	1	1	1	1	1
	32	1	1	1	1	1	1	1	1	1	1
	33	1	1	1	1	1	1	1	1	1	1
	34	1	1	1	1	1	1	1	1	1	1
	35	1	1	1	1	1	1	1	1	1	1
	36	1	1	1	1	1	1	1	1	1	1
